# Conceptual Design for Developing a Bioreactor with Nano-materials for the Triclosan Removal in Wastewater Treatment Systems

**Published:** 2018-01

**Authors:** Do Gyun LEE

**Affiliations:** Dept. of Environmental Engineering, Incheon National University, Incheon, 22012, South Korea

## Dear Editor-in-Chief

Triclosan (5-chloro-2-(2, 4-dichlorophenoxy) phenol) has been widely used as a synthetic broad-spectrum antimicrobial agent in industrial products and personal care products (1). However, due to the release of triclosan into aquatic environment thorough wastewater effluents, concerns about triclosan detection includes the potential risk to promote the development of cross-resistance to antibiotics, the potential toxicity of triclosan itself and its metabolites, such as carcinogenicity and endocrine disruption, on human and ecological health (2, 3).

Recently, U.S. FDA (Food and Drug Administration) issued a rule banning the use of triclosan in antibacterial hand and body soaps. A bioreactor design in a wastewater treatment plant is one of important elements for successful application of biological treatment process. Since the conventional bioreactor containing activated sludge (mixed cultures) is typically designed for the non-selective removal of contaminants present in the bioreactor, a new design approach for bioreactor is needed to intensively remove a target pollutant, such as triclosan. In addition, the significant outcomes and innovated solutions have been established along with nanomaterial development and applications for the water treatments (4).

Thus, it is needed to apply nanotechnology with a novel design of a bioreactor to a wastewater treatment system to achieve the higher removal efficiency of a target pollutant (i.e. triclosan). To develop successfully this novel bioreactor system with nanomaterials, the major points below should be taken into consideration.

The types of nanomaterials are an important factor for the removal of triclosan due to the exceptional physical, mechanical, and electronic properties of nanomaterials. Thus, carbon-based nanomaterials and zeolite materials would be potential candidates to be used for triclosan removal in a bioreactor. The carbon-based nanomaterials can be suitable for the adsorbents and bacterial growth materials in natural and engineered environmental systems (5). While the traditional carbon sorbents have limited absorbed capacity due to the density of surface active sites, the activation energy of sorption bonds, and slow kinetics and equilibrium condition, nanostructural carbon-based materials provides the high adsorption capacity, rapid equilibrium rates, and broad pH range resistance as well as the consistency with Langmuir or Freundlich isotherms (6). In addition, zeolites are aluminosilicates and well known for their unique characteristics such as high surface area and the bonding abilities of specific functional groups. The nano-scale zeolites have the increased surface areas and decreased diffusion path lengths, resulting in the higher reactivity of active sites and improvement of their catalytic properties (6). Furthermore, it is important to select an effective nanostructure material support that is suitable for the attachment/growth of bacteria and delivery of nutrients in a bioreactor system.

A novel configuration of bioreactor containing triclosan-degrading bacteria should be considered to provide more surface area to contact a target compound (i.e., triclosan). A design of the bioreactor with separate narrow flow channels, made of carbon-based nanomaterials or zeolites and have triclosan-degrading microorganisms on their surface, could be one of alternatives. Since nanomaterials can provide extremely vast surface area for bacteria growth, fixed triclosan-degraders on the surface of nanomaterials would have higher chance to be exposed to triclosan molecules than the randomly dispersed bacteria in the bioreactor, resulting in the high removal efficiency of triclosan. Investigation of the predominance of bacterial community, biofilm growth morphology, bacterial growth rate on nanomaterials, and the interaction between nanomaterials and triclosan-degrading microorganisms should be taken into account. Furthermore, the investigation of possible removal mechanisms by the attached biofilm layers of triclosan-degrading microorganisms should be considered.

This article aimed to suggest a conceptual approach for developing a target pollutant-specific bioreactor incorporated with nanomaterials to improve the removal efficiency of triclosan in wastewater treatment. Consequently, less or none-discharge of triclosan from wastewater effluents into receiving waters make our aquatic environment more healthy and sustainable.
